# A Model for Spheroid versus Monolayer Response of SK-N-SH Neuroblastoma Cells to Treatment with 15-Deoxy-*PGJ*_2_

**DOI:** 10.1155/2016/3628124

**Published:** 2016-12-01

**Authors:** Dorothy I. Wallace, Ann Dunham, Paula X. Chen, Michelle Chen, Milan Huynh, Evan Rheingold, Olivia Prosper

**Affiliations:** ^1^Department of Mathematics, HB 6188, Dartmouth College, Hanover, NH 03768, USA; ^2^Department of Mathematics, University of Kentucky, Lexington, KY 40506, USA

## Abstract

Researchers have observed that response of tumor cells to treatment varies depending on whether the cells are grown in monolayer, as* in vitro* spheroids or* in vivo*. This study uses data from the literature on monolayer treatment of SK-N-SH neuroblastoma cells with 15-deoxy-*PGJ*_2_ and couples it with data on growth rates for untreated SK-N-SH neuroblastoma cells grown as multicellular spheroids. A linear model is constructed for untreated and treated monolayer data sets, which is tuned to growth, death, and cell cycle data for the monolayer case for both control and treatment with 15-deoxy-*PGJ*_2_. The monolayer model is extended to a five-dimensional nonlinear model of* in vitro* tumor spheroid growth and treatment that includes compartments of the cell cycle (*G*_1_, *S*, *G*_2_/*M*) as well as quiescent (*Q*) and necrotic (*N*) cells. Monolayer treatment data for 15-deoxy-*PGJ*_2_ is used to derive a prediction of spheroid response under similar treatments. For short periods of treatment, spheroid response is less pronounced than monolayer response. The simulations suggest that the difference in response to treatment of monolayer versus spheroid cultures observed in laboratory studies is a natural consequence of tumor spheroid physiology rather than any special resistance to treatment.

## 1. Introduction

Cancer therapies are tested thoroughly on monolayer layers to identify not only their effectiveness but also the specific manner in which they impede cell division or induce apoptosis. It is understood that the effectiveness of treatment in monolayer does not predict equivalent effectiveness* in vivo*. However, tumor spheroids cultured* in vitro* are considered somewhat similar to small nodal tumors in a preangiogenic state [1]. Both cases have actively proliferating cells near a nutrient source, quiescent cells farther from that source, and necrotic cells at a farther distance from nutrient. Spheroids grown* in vitro* are probably a better predictor of therapeutic response of small nodal tumors than* in vitro* monolayer layers.

As part of a study on metronomic therapy of breast cancer, Klement et al. [2] published three examples of increasing concentrations of treatments on resistant cell lines, comparing the effect on monolayer versus spheroid cultures. Their experiments included three treatments, adriamycin, vinblastine, and cisplatinum, on resistant cell lines MD22, MVB9, and CDDP-S4. Their results showed a clear discrepancy in response to treatment, with monolayer cultures showing a substantial growth reduction in response to increased concentrations of each of the three treatments, while spheroid cultures showed a far less marked decrease in growth in response to the same concentrations. As an example of this phenomenon, when sufficient vinblastine was applied to monolayers of MVB9 to reduce proliferating cells at 24 hours to around 20% of the untreated monolayer, the same concentration applied to the spheroid culture reduced the spheroid to approximately 80% of the untreated spheroid after 72 hours. This pattern held for the other cell lines as well [2]. As the experiments terminated after only a few days, it is not known whether either culture would be completely killed by continued treatment.

The results of this paper raise a few questions which may be approached through simulations. The first is whether it is likely that the results in Klement et al. [2] would be replicated with other cell lines and other treatments. In particular, that study used cell lines which were known to be resistant to the treatments applied. It would be useful to know whether to expect a similar result with tumor cell lines that are not particularly resistant to a given treatment. One should also ask to what extent this differential response to treatment is a natural consequence of tumor spheroid physiology. Unlike monolayers, spheroids exhibit a tripartite anatomy of proliferating, quiescent, and necrotic cells [3]. Unlike monolayers, spheroids spontaneously cease growth [4]. Perhaps the physiological processes inherent in spheroid development provide a natural protection against certain therapies. A model of spheroid growth and response to therapy would allow* in silica* experiments that answer these questions and would be a useful predictor for therapeutic response of preangiogenesis* in vivo* tumor nodes.

The spontaneous cessation of tumor spheroid growth was conjectured to be due to the inability of nutrients to penetrate to the core of the spheroid, which subsequently undergoes necrosis [4]. The limits of diffusion, however, do not rule out the existence of large spheroids with a small outer layer of proliferating cells, thin enough to receive nutrients. Numerical experiments confirm that diffusion of nutrients alone is insufficient to explain cessation of growth [5, 6]. Further* in vitro* experiments show that the necrotic core produces tumor necrosis factors that inhibit proliferation [7]. A specific factor, known as TNF-*α*, has been shown to induce apoptosis in actively proliferating cells [8, 9]. Several tumor spheroid models, incorporating tumor necrosis factors as a source of apoptosis of proliferating cells, produce* in silica* spheroids with qualitatively correct development [10]. In these models overall spheroid growth ceases without resorting to an artificial restraint. That is, growth ceases because of the interference of tumor necrosis factor in these models, and when that factor is removed growth does not cease. Furthermore, these models exhibit a range of behaviors consistent with qualitative observations of* in vitro* spheroids [4, 7].

To make sense of how model simulations can reflect therapies, it is necessary to tune general models with good qualitative behavior to the specifics of a particular cell line and therapy. This paper considers treatment of SK-N-SH neuroblastoma cells with 15-deoxy-*PGJ*_2_. Kim et al. [11] conduct a series of experiments of increasing concentrations of 15-deoxy-*PGJ*_2_ on monolayer layers. They concluded that the treatment both inhibits proliferation at the *G*_2_/*M* stage and induces apoptosis at that stage. Data for both the untreated and treated cell lines are given in that paper. Data for untreated spheroids of the same cell line is given in Carlsson et al. [1].

The data published in these two papers [1, 11] and the general models for spheroid growth [10] are the basis for developing a more complex spheroid model that incorporates cell cycle dynamics. It preserves the qualitative behaviors observed in spheroids [4, 7], while tuning to cell cycle dynamics measured in [11] and spheroid growth dynamics measured in [1]. Therapeutic parameters derived from monolayer experiments of Kim et al. are then applied to the spheroid growth model, and the results are compared with the response of monolayers.

## 2. Analysis

**Figure 1:**
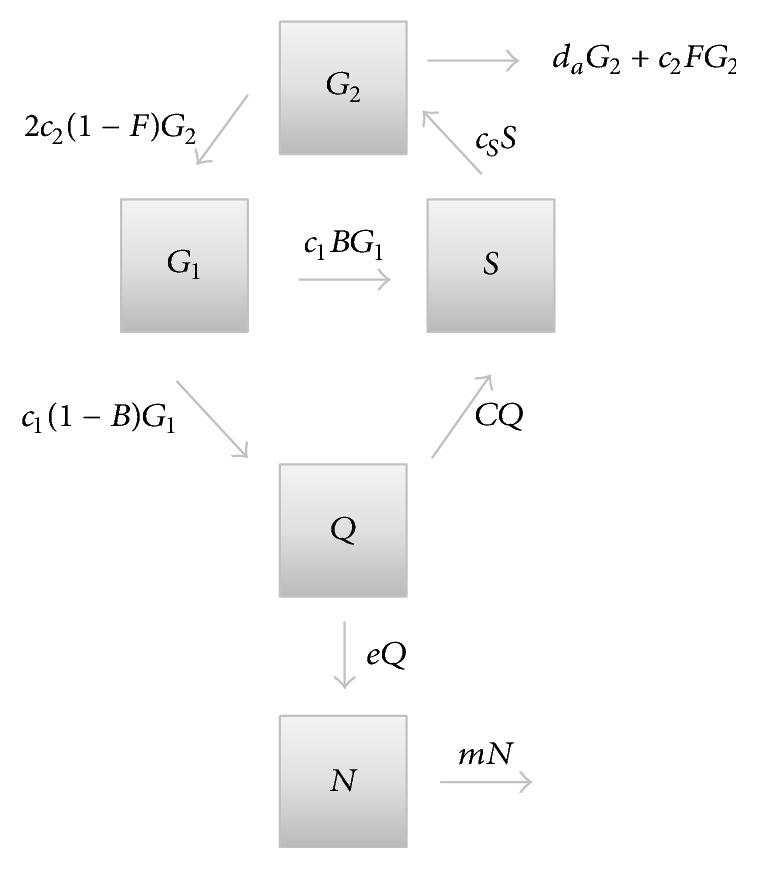
A cell cycle model (compartments *G*_1_, *S*, and *G*_2_) is extended to include spheroid dynamics, including quiescent cells (*Q*) and the necrotic core (*N*). Rates are indicated near arrows. Quantities *B*, *C*, and *F* are dependent on the state of the system. If parameters are chosen so that *F* = 0 and *B* = 1, the resulting model simulates monolayer growth.

The nonlinear dynamic model developed here includes five compartments, *G*_1_, *S*, *G*_2_, *Q*, and *N*. The first three of these, *G*_1_, *S*, and *G*_2_, correspond to the stages of the cell cycle. The cell cycle leads to equations that include four quantities of the same name, *G*_1_, *S*, *G*_2_, and *M*. However the data in the literature tends to combine measurements for *G*_2_ and *M*, so in the model the compartments for *G*_2_ and *M* are combined into just one compartment, *G*_2_. To reflect spheroid anatomy, quiescent cells, *Q*, are an additional compartment. The dead necrotic core, *N*, must be included because it is part of the measurement of total spheroid size and because it has an effect on proliferation through TNF-*α*. The compartment model is illustrated in Figure 1.

### 2.1. A Tuned Linear Model of Monolayer Growth

Monolayer layers exhibit exponential growth, at least in the short run, and the cell cycle for such cultures is modeled by a system of ordinary differential equations given below.

The rate of change of *G*_1_ is given by transition (upon doubling) from *G*_2_ minus transition into *S*:

(1) $$\begin{array}{c} G_{1}^{\prime }=2c_{2}G_{2}-c_{1}G_{1}. \end{array}$$

The rate of change of *S* is given by transition from *G*_1_ to *S* minus transition into *G*_2_:

(2) $$\begin{array}{c} S^{\prime }=c_{1}G_{1}-c_{S}S. \end{array}$$

The rate of change of *G*_2_ is given by transition from *S* to *G*_2_ minus transition (upon doubling) into *G*_1_ and minus some death rate:

(3) $$\begin{array}{c} G_{2}^{\prime }=c_{S}S-c_{2}G_{2}-d_{a}G_{2}. \end{array}$$

Writing the system as *X*′ = *AX*, we expect solutions of the form

(4) $$\begin{array}{c} X=e^{\lambda t}\left(\begin{array}{c} g_{1}^{*}\\ s^{*}\\ g_{2}^{*} \end{array}\right). \end{array}$$

Here *X* ^*∗*^ satisfies *AX* ^*∗*^ = *λX* ^*∗*^, where *λ* = ln⁡(2)/*D* and *D* is the doubling time for the culture. The numbers (*g*_1_^*∗*^, *s* ^*∗*^, *g*_2_^*∗*^) are the proportions of cells in each stage in the limit. As the cultures used in the monolayer experiments are not synchronized, it is also the proportion of cells in each stage at the start of the run, more or less. Plate experiments generally indicate a death rate for the control (untreated) culture, allowing us to deduce *d*_*a*_, or natural death due to apoptosis.

The equation *AX* ^*∗*^ = *λX* ^*∗*^ leads to three linear equations that can be solved in terms of experimentally derived quantities, (*g*_1_^*∗*^, *s* ^*∗*^, *g*_2_^*∗*^, *d*_*a*_, *λ*), to give

(5) c2=λ+dag2∗g2∗,c1=2c2g2∗−λg1∗g1∗,cS=c1g1∗−λs∗s∗.

These equations were tuned to monolayer data in Kim et al. [11].

Using the Routh-Hurwitz Criterion, we determined that the monolayer dies out when *c*_2_ < *d*_*a*_ and persists, otherwise. This can be easily shown by noting that the characteristic equation for the linear system is *λ* ^3^ + *a*_2_ *λ* ^2^ + *a*_1_ *λ* + *a*_0_ = 0, where *a*_0_ = *c*_1_ *c*_*S*_(*d*_*a*_ − *c*_2_), *a*_1_ = *c*_1_ *c*_*S*_ + *c*_1_(*c*_2_ + *d*_*a*_) + *c*_*S*_(*c*_2_ + *d*_*a*_), and *a*_2_ = *c*_1_ + *c*_*S*_ + (*c*_2_ + *d*_*a*_). The Routh-Hurwitz Criterion states that all roots of the characteristic polynomial have negative real part if and only if *a*_0_, *a*_1_, *a*_2_ > 0, and *a*_2_ *a*_1_ > *a*_0_. The last three inequalities are immediately satisfied, and *a*_0_ > 0 if and only if *c*_2_ < *d*_*a*_. Consequently, *c*_2_/*d*_*a*_ = 1 represents a threshold between extinction and persistence of the monolayer.

#### 2.1.1. Tuning the Control (Untreated) Monolayer Model to Data

Table 1 gives constants derived from data on monolayers of neuroblastoma cell line SK-N-SH. Doubling time was not given in the main source paper [11] and was taken as an average of several given in the literature [1, 12–16]. Equilibrium proportions, (*g*_1_^*∗*^, *s* ^*∗*^, *g*_2_^*∗*^), of cells in each stage of the cell cycle, were taken from the control run in Kim et al. [11], with a slight adjustment so that they sum to 100%. As reported in the same study, *d*_*a*_ was taken to be 0 for the control run.

#### 2.1.2. Modeling Treatments on Monolayers

The experiments described in Kim et al. [11] include data on treatments of 15-deoxy-*PGJ*_2_ at various concentrations, applied to the SK-N-SH cell line. The authors conclude that 15-deoxy-*PGJ*_2_ acts both to arrest cells in the *G*_2_/*M* stage and to induce apoptosis at that stage. These actions correspond to a change in parameters *c*_2_ and *d*_*a*_ in the cell cycle model. These parameters were adjusted to give a local best match with data for the experiments described by optimizing total percent error in *G*_1_, *S*, and *G*_2_ and mortality over a reasonable range. The resulting simulated data is in Table 2 along with the reported measured data for comparison purposes. The match of simulation to data was better for the lower doses. Initial conditions were set to a total volume of 1, with proportions given by (*G*_1_(0), *S*(0), *G*_2_(0)) = (*g*_1_^*∗*^, *s* ^*∗*^, *g*_2_^*∗*^).

### 2.2. A Spheroid Model Incorporating the Cell Cycle

Wallace and Guo [10] describe a class of models for spheroid growth that consider proliferating, quiescent, and necrotic cells. Various versions of these models were tested against a range of qualitative observations [4, 7]. In order to preserve the features of the cell cycle model given earlier, the proliferating compartment in those earlier models is replaced with the entire cell cycle, and simple transitions between proliferating and other compartments are replaced with transitions that depend on relative availability of nutrient or presence of TNF-*α*. The goal was to revise and extend the models in [10] to include cell cycle dynamics, while maintaining consistency with qualitative observations of spheroid growth and simultaneously matching the data from [1]. A compartment diagram is pictured in Figure 1.

The path to enter the quiescent state, *Q*, is assumed to be taken by some cells at the *G*_1_ stage as an alternative to entering *S*. The return from quiescent to nonquiescent, which has been observed [17], is assumed to move the cell into the *S* stage. The effect of necrosis on proliferating cells is assumed to occur at the division stage via apoptosis [8, 9]. These assumptions, pictured in Figure 1, yield the following equations. All units are in volume, 10^6^ *μ*m^3^.

The rate of change of *G*_1_ is given by transition (upon doubling) from *G*_2_, with fraction *F* undergoing apoptosis due to TNF-*α* and fraction 1 − *F* entering *G*_1_, minus transition out of *G*_1_ with fraction *B* entering *Q* and 1 − *B* entering *S*:

(6) $$\begin{array}{c} G_{1}^{\prime }=2c_{2}\left(\;1-F\;\right)G_{2}-c_{1}\left(\;B\;\right)G_{1}-c_{1}\left(\;1-B\;\right)G_{1}. \end{array}$$

The rate of change of *S* is given by transitions from *G*_1_ and *Q*, minus transition out of *S* into *G*_2_:

(7) $$\begin{array}{c} S^{\prime }=c_{1}\left(\;B\;\right)G_{1}+CQ-c_{S}S. \end{array}$$

The rate of change of *G*_2_ is given by transitions from *S*, minus transition out of *G*_2_ with fraction *F* undergoing apoptosis due to TNF-*α*, fraction 1 − *F* entering *G*_1_, and natural or therapy-induced death rate *d*_*a*_:

(8) $$\begin{array}{c} G_{2}^{\prime }=c_{S}S-c_{2}\left(\;1-F\;\right)G_{2}-c_{2}FG_{2}-d_{a}G_{2}. \end{array}$$

The rate of change of *Q* is given by transition from *G*_1_ with fraction *B* entering *Q*, minus transitions out of *Q* either returning to *S* or dying and entering *N*:

(9) $$\begin{array}{c} Q^{\prime }=c_{1}\left(\;1-B\;\right)G_{1}-CQ-eQ. \end{array}$$

The rate of change of *N* is given by death of cells in *Q*, minus dissolution of material in *N*:

(10) $$\begin{array}{c} N^{\prime }=eQ-mN. \end{array}$$

When *B* = 1 and *C* = *F* = 0, these equations are identical for those of the cell cycle, producing a mass of proliferating cells undergoing exponential growth. For the new case of a spheroid, *B* and *F* are taken to be functions describing the dependency of transition processes on availability of nutrient, in the case of *B*, or amount of necrosis, in the case of *F*. The parameter *C* depends on the availability of nutrient to *Q*, which is blocked by the uptake of the nonquiescent compartments *G*_1_, *S*, and *G*_2_. For convenience a new variable, *T*, describes the sum of all compartments in the model, and a second variable, *P*, describes the live, nonquiescent cells (*P* = *G*_1_ + *S* + *G*_2_). Extra apoptosis due to treatment, *d*_*a*_ *G*_2_, is assumed to occur at the transition from *G*_2_ to *G*_1_ as in the linear model.

The passage of *G*_1_ to *S* in the linear model is controlled by the constant *c*_1_, which in the extended model is taken to be the maximum rate of transfer between these compartments, as some of the *G*_1_ cells are sent instead to the *Q* compartment. The function *B* must range therefore between 0 and 1. Nutrients, including oxygen, are assumed to enter the cell at a spherical boundary, so for a fixed amount of *G*_1_ the function *B* should increase to 1 or decline to 0 with surface area proportional to *T* ^2/3^. As the proportion of proliferating cells increases relative to surface area, *B* should decline to zero, allocating more cells to the *Q* compartment. A class of functions that behaves this way is given by

(11) $$\begin{array}{c} B=\frac{T^{2/3}}{s_{1}+G_{1}+T^{2/3}}. \end{array}$$

The return from the quiescent state to the proliferating state would occur for some fraction of cells when nutrients cease to be blocked by the nonquiescent cells, *P*. Little data is available on this process, although it has been observed to happen when hypoxic conditions are relieved [17]. Here the process is modeled by a function of *P* and surface area proportional to *T* ^2/3^, which is assumed to have a maximum rate of *c*_*q*_ when surface area is large compared to *P* and which approaches a minimum rate of 0 when surface area is small compared to *P*.

A class of functions that behaves this way is given by

(12) $$\begin{array}{c} C=c_{q}\frac{T^{2/3}}{s_{q}+P+T^{2/3}}. \end{array}$$

Both functions *B* and *C* have the property that they go to zero as overall spheroid size goes to zero. This is not a biologically reasonable behavior, as no cells would be expected to become quiescent at very small spheroid size. A more biologically reasonable function would be given by *s*_1_ = 0, but then numerical difficulties could occur at small values. The given formulation of the functions *B* and *C* works well for spheroid sizes greater than 1. As the model is designed for spheroids on the order of 10^6^ *μ*m^3^ the effect near zero is easily avoided for small *s*_1_ and *s*_*q*_. Both *s*_1_ and *s*_*q*_ have been set to 0.0001.

Finally, the fraction of cells at the *G*_2_ stage that reproduce or die is determined by the presence of TNF-*α* [8, 9] which is assumed to be present in proportion to the amount of necrotic tissue, *N*. The maximum rate at which apoptosis could occur is assumed to be less than the intrinsic rate *c*_2_ at which that stage of the cell cycle proceeds. A class of functions describing this choice is given by

(13) $$\begin{array}{c} F=\frac{N}{s_{n}+N}. \end{array}$$

These equations were tuned to spheroid culture data in Carlsson et al. [1], while fixing the constants derived from monolayer data.

#### 2.2.1. Tuning the Control (Untreated) Spheroid Culture Model to Data

Cell cycle parameters *c*_1_, *c*_*s*_, *c*_2_, and *d*_*a*_ were kept the same as for control monolayer data described in Table 1. Spheroid diameters over time are reported as a figure in Carlsson et al. [1].  Table 3 gives estimated values read from that table. Spheroid volumes were computed from diameter data in Table 3, reported in Table 4. Although this paper does not give cell cycle data, it does report a monolayer doubling time of 40–50 hours, consistent with the average used in Table 1. On the last day of the experiment, the thickness of the viable cell rim was recorded via light microscopy. More than twenty spheroids were tested, and clearly there was a lot of variation in this number. Table 4 gives the volumes of live and necrotic compartments computed from the data in Table 3.

The two most difficult parameters to identify from data are *s*_1_ and *s*_*q*_, which describe the functional response of proliferating and quiescent cells to the presence of nutrient. Although it plays an important role in spheroid physiology, the quiescent compartment, *Q*, is difficult to measure without explicit cell cycle data. It is not measured in the study to which this model is tuned. A simpler assumption would be that the functional response is given by a direct proportion, with *s*_1_ = *s*_*q*_ = 0. Instead, an arbitrary choice of *s*_1_ = *s*_*q*_ = 0.0001 gives a functional response that is almost a direct proportion, while avoiding any numerical difficulties that could arise if the denominator were to get small. This choice was made after some experimentation and still gave a decent match with data.

Four parameters remain to be identified. Of these, two (*m*, *s*_*n*_) control the relative size of the live versus dead compartments. Summing (6) through (10) at equilibrium yields the relation

(14) $$\begin{array}{c} G_{2}^{*}=mc_{2}^{-1}\frac{N^{*}\left(s_{n}+N^{*}\right)}{s_{n}-N^{*}}. \end{array}$$

Numerical exploration indicates that the two parameters that appear in this equation, *m* and *s*_*n*_, do indeed have the biggest effect on the final values of *N* and *T* − *N*. In order to have a positive quantity of proliferating cells at equilibrium, *s*_*n*_ must be greater than *N* ^*∗*^. Experimentally, the highest volume of the spheroid is about 36(*∗*10^6^ *μ*m^3^). Starting with a value of approximately twice that, *m* and *s*_*n*_ were adjusted to give, in order of priority, a final volume at *t* = 31 (29 days) close to 36, with live and dead cell volumes in the reported ranges.

Two parameters remain, *c*_*q*_ and *e*, describing transition rates from *Q* to *S* and from *Q* to *N*, respectively. As *Q* is not measured directly, the impact of these parameters is strictly visible in the total growth pattern. More cells in the proliferating compartments will create faster growth. To fit these, a MATLAB best fit program was used on the growth data for total volume over time. Fitted parameters are reported in Table 4.

In addition to matching data, overall long-term behavior of the model was compared to qualitative observations and was found to behave well. Initial conditions for all data matching and treatment simulations were set by the volume of the first data point in [1], assuming that all cells are in the proliferating compartment (*Q*(0) = 0, *N*(0) = 0), distributed in the ratio given by (*G*_1_(0), *S*(0), *G*_2_(0)) = (*g*_1_^*∗*^, *s* ^*∗*^, *g*_2_^*∗*^).

#### 2.2.2. Simulation of Treatments on Spheroids

With all parameters specified for the model of an untreated SK-N-SH spheroid, it only remains to alter those parameters corresponding to treatment with 15-deoxy-*PGJ*_2_. The parameters involved, *c*_2_ and *d*_*a*_, were set according to their derived values in Table 2. The linear monolayer model was run for 24 hours whereas the spheroid model was run for 72 hours in order to see a bigger effect. The study by Klement et al. [2] showed a noticeable difference in response between monolayer and spheroid cultures. This response was measured by a methyl-[3H]-thymidine incorporation assay, which labels cells in the *S* phase of the cell cycle [18]. This measurement is sometimes considered a proxy for the proliferating compartment.  Figure 6 shows relative effect sizes for the 15-deoxy-*PGJ*_2_ concentrations studied in Kim et al. [11].

#### 2.2.3. Bifurcation Analysis of Spheroid Model

To determine the impact of treatment intensity on spheroid volume and persistence of the spheroid, we performed a bifurcation analysis of the spheroid model. In particular, we calculated numerically (using the MATLAB function “lsqnonlin”) the volume of proliferating, quiescent, and necrotic cells at equilibrium as a function of *d*_*a*_, the rate of *G*_2_ apoptosis, for choices of *c*_2_ corresponding to the five treatment intensities, *c*_2_ and *c*_2,new_ = .001*c*_2_, 0.19*c*_2_, 0.29*c*_2_, 0.47*c*_2_.

## 3. Results

### 3.1. Monolayer Models

**Figure 2:**
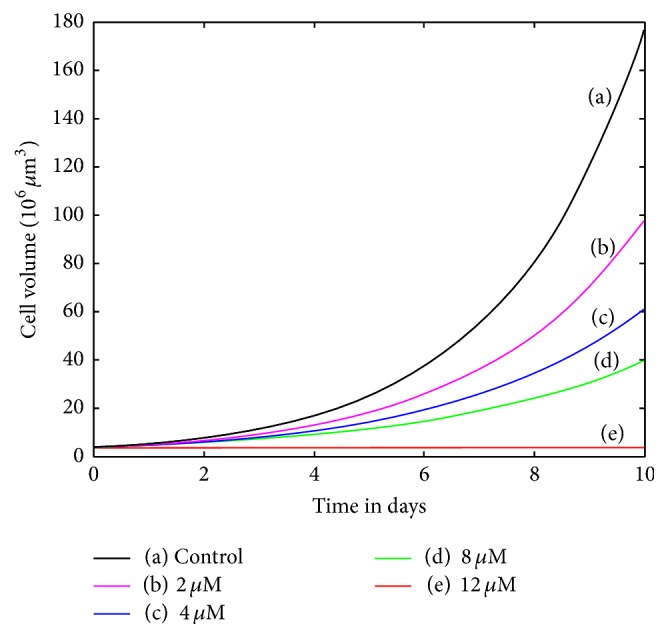
Simulated monolayer treatment of SK-N-SH neuroblastoma cells with 15-deoxy-*PGJ*_2_ at varying concentrations. Curves are labeled by the value of *c*_2_, with keys as follows: (control, *c*_2_ = 3.85, *d*_*a*_ = 0), (2 *μ*M, *c*_2,new_ = 0.47*c*_2_, *d*_*a*,new_ = 0.60), (4 *μ*M, *c*_2,new_ = 0.29*c*_2_, *d*_*a*,new_ = .59), (8 *μ*M, *c*_2,new_ = 0.19*c*_2_, *d*_*a*,new_ = .87), and (12 *μ*M, *c*_2,new_ = 0.001*c*_2_, *d*_*a*,new_ = 1.3).

**Figure 3:**
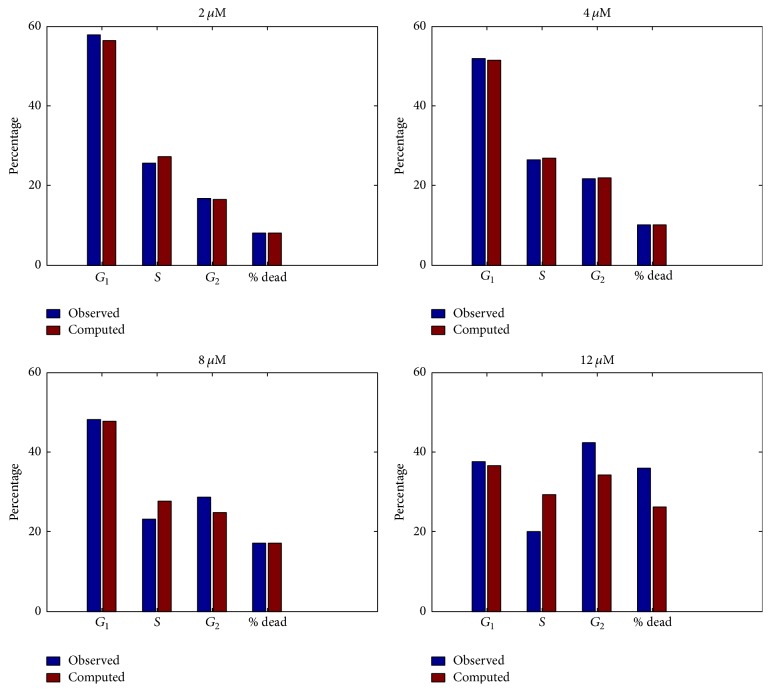
Model simulation of cell cycle analysis after 24 hours of treatment, versus measured values. Data is taken from [11]. The model matches data well at low concentrations, with a poor match at 12 *μ*M. Mean squared error for 2, 4, and 8 *μ*M treatments all together is 3.5 percentage points. MSE for all treatments is 18 percentage points.

Rapidly growing tumor monolayers exhibit characteristic doubling times, death rates, and cell cycle proportions that are enough to determine a linear model completely, leading to simple algebraic expressions for all parameters. Data for treated monolayers are another matter. In the example studied here, two parameters, *c*_2_ and *d*_*a*_, corresponding to researchers' best understanding of the action of 15-deoxy-*PGJ*_2_ on SK-N-SH cell lines were altered in an effort to match treatment intensities of 2, 4, 8, and 12 *μ*M concentrations, as in Figure 2. A good fit was obtained for the low concentrations (2 and 4 *μ*M). At 8 *μ*m, the mortality rate was matched well with some sacrifice of accuracy for cell cycle proportions. At 12 *μ*M the cell cycle proportions produced by the model did not match the data particularly well, as seen in Figure 3. Mean squared error for 2, 4, and 8 *μ*M treatments altogether is 3.5 percentage points. MSE for all treatments is 18 percentage points. This suggests that, at high concentrations, other transitions are being affected besides the *G*_2_/*M* to *G*_1_ transition.

### 3.2. Spheroid Models

**Figure 4:**
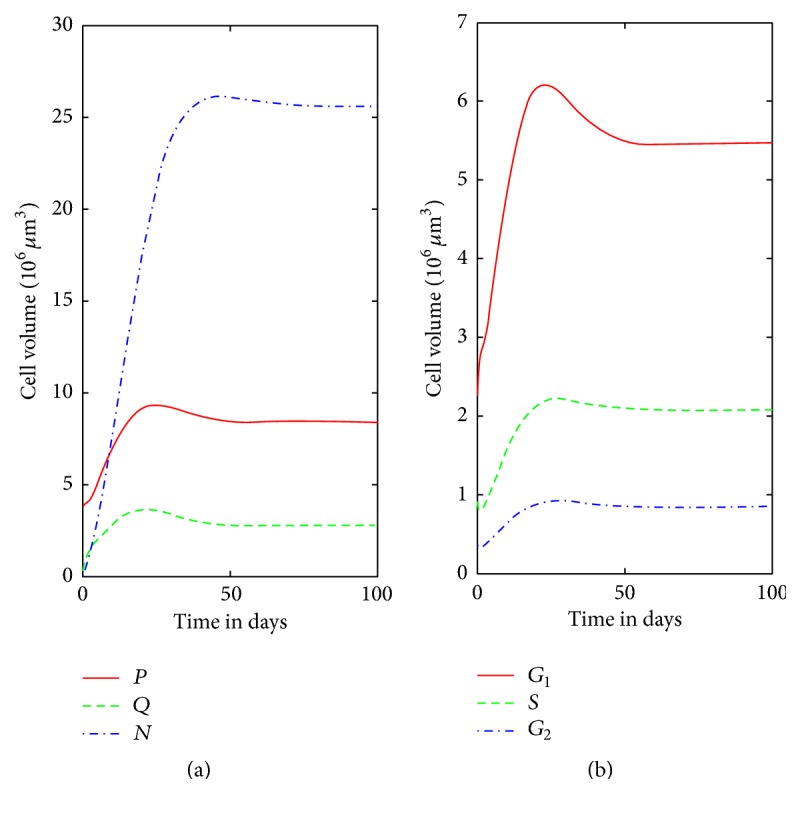
Model predictions for spheroid growth to 100 days. (a) Relative sizes of proliferating (*P*), quiescent (*Q*), and necrotic (*N*) compartments. (b) Breakdown of the proliferating compartment into parts of the cell cycle on the right.

**Figure 5:**
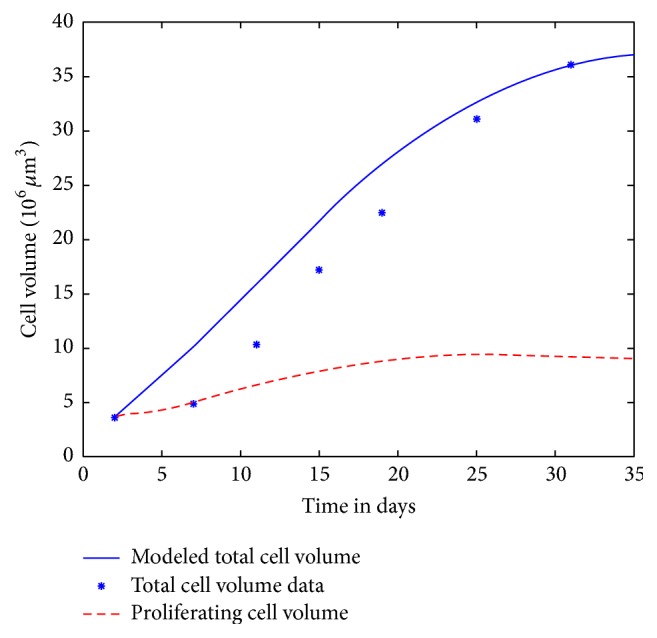
The SK-N-SH neuroblastoma spheroid model with default (control) parameters given in Tables 1 and 4. Total spheroid size is graphed, along with the data from Carlsson et al. [1]. The proliferating compartment is also graphed. Note that day 0 corresponds to the initial conditions given as day 2 in the data; similarly day 29 in the image corresponds to day 31 in the data set taken from [1]. Note that priority was given to getting a good fit for both *T* and *P* at the final data point, with some sacrifice of accuracy at intermediate points. Table 4 gives numerical data and model prediction for the last time data.

It is possible to extend the linear model of the cell cycle in monolayer growth to a nonlinear model of spheroid growth, as illustrated in Figure 1. Nonlinearity is included in the transition rates between proliferating to quiescent cells and in the action of TNF-*α* in inducing apoptosis at the *G*_2_ stage. All transition rates in this model are bounded by those determined by monolayer cell cycle data, with the exception of the transition from quiescent to proliferating, which was not measured. The model was tuned to a specific cell line and treatment, giving growth curves as in Figure 4. The model produces a spheroid that stops growing, has a necrotic core, and whose proliferating compartment remains alive; qualitative observations are reported in the literature [4, 7]. The tuned model fits the initial and final data quite well but underestimated the intermediate measurements, as seen in Figure 5.

### 3.3. Treatment of Spheroids versus Monolayers

**Figure 6:**
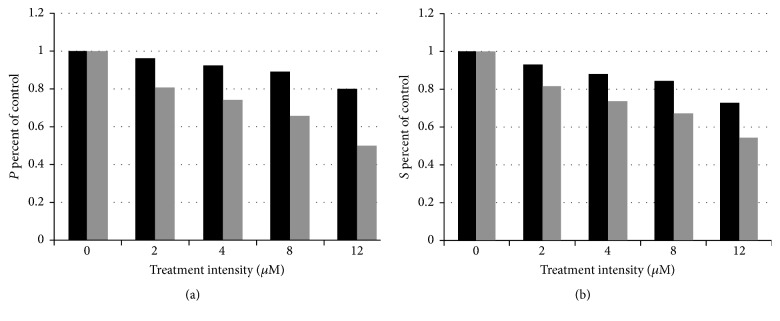
Response of SK-N-SH neuroblastoma spheroid and monolayer models to increasing 15-deoxy-*PGJ*_2_ treatment concentration, measured in micromolar concentrations as in [11]. Response of spheroid model (black) is given as percent of control after 3 days of growth. Response of monolayer model (gray) is given as percent of control after one day of growth.

**Figure 7:**
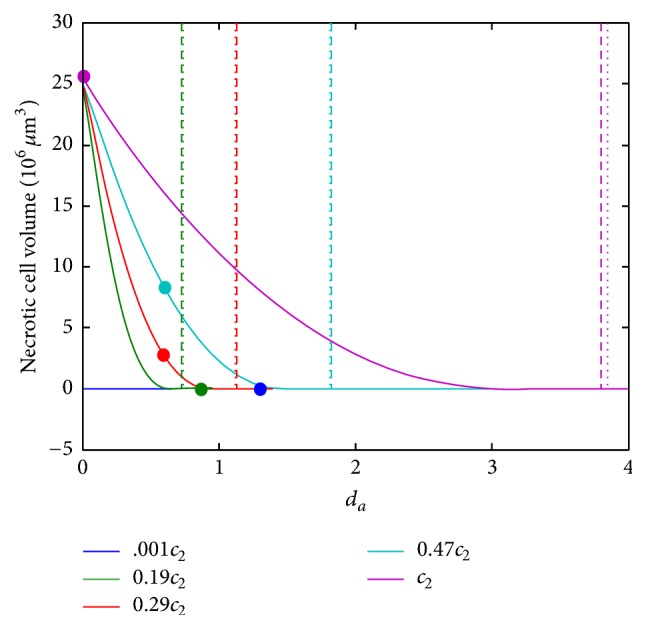
Bifurcation diagram for equilibrium of necrotic cells in spheroid model. Each line represents the equilibrium volume of cells at a different treatment intensity *c*_2,new_, as a function of *d*_*a*_, in units of 10^6^ *μ*m^3^. Filled circles denote the equilibria using the fitted pair of parameters (*c*_2,new_, *d*_*a*,new_) in Table 2 and Figure 2 (control, *c*_2_ = 3.85, *d*_*a*_ = 0), (2 *μ*M, *c*_2,new_ = 0.47*c*_2_, *d*_*a*,new_ = 0.60), (4 *μ*M, *c*_2,new_ = 0.29*c*_2_, *d*_*a*,new_ = .59), (8 *μ*M, *c*_2,new_ = 0.19*c*_2_, *d*_*a*,new_ = .87), and (12 *μ*M, *c*_2,new_ = 0.001*c*_2_, *d*_*a*,new_ = 1.3). Vertical lines denote critical values for the monolayer model (*d*_*a*_ = *c*_2_). At values of *d*_*a*_ below these critical values the monolayer grows exponentially. For values higher than this it declines exponentially. Diagrams for the proliferating and quiescent compartments (omitted here) are similar to this diagram. Note that equilibrium values for all spheroid compartments drop to near zero before the critical value for the monolayer model.

**Figure 8:**
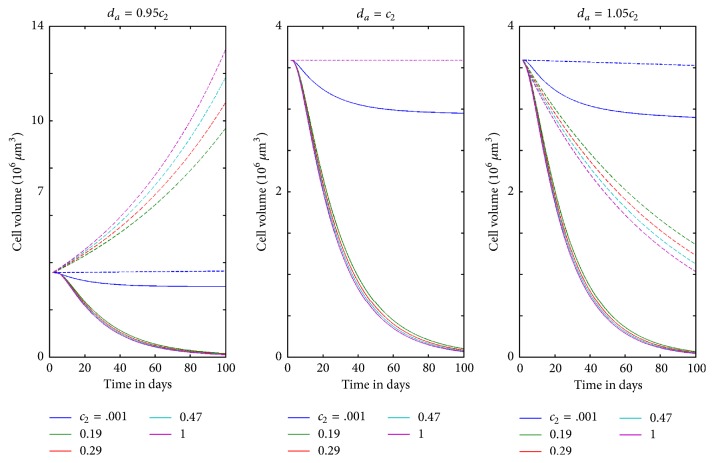
Long-term behavior of SK-N-SH neuroblastoma spheroid, under hypothetical continued treatment, at values of *d*_*a*_ near the critical threshold for the monolayer model. Dashed lines give the behavior for the monolayer model and solid lines give the behavior of the spheroid model. For purposes of comparison, the initial conditions are given by the data set taken from [1], for both the spheroid and monolayer models. Colors represent the five choices of *c*_2_ corresponding to the control and four treatment intensities. Note that a small change in *d*_*a*_ creates a substantial response in the monolayer model, while the spheroid dynamics are not much different.

The purpose of constructing these models was to simulate the different response to treatment between monolayer and spheroid cultures.  Figure 6 illustrates this effect. Simulations of increased concentrations of 15-deoxy-*PGJ*_2_ on SK-N-SH spheroids produced noticeably less effect than on monolayers, even when spheroids are treated for 72 hours instead of 24 hours. In this particular example, monolayers are still growing in size at 2 and 4 micromolar concentrations, but at a greatly reduced rate. At 8 *μ*M, the colony size of a monolayer decreases. By contrast, for spheroids, the total volume is finite and is reduced by all treatments. However, the effect size is small. Even though the total volume is reduced, the spheroid may persist.  Figure 7 shows a numerically computed bifurcation analysis for the spheroid model, with vertical lines marking critical values in the monolayer model. The diagram indicates that a lower intensity of treatment may suffice to drastically reduce the size of a spheroid, as opposed to a monolayer. However the effect of short-term treatment (2-3 days) may produce a less marked response in the spheroid, as in Figure 6.  Figure 8 shows the effect of treatment sustained over a long period, for parameters near the critical value for the monolayer model. Although a therapy with these parameters may not be biologically feasible, Figure 8 highlights the difference in dynamics over a longer period.

## 4. Discussion

The behavior of the monolayer model under treatment conditions is consistent with the conclusions in [11] that treatment blocked the *G*_2_ to *G*_1_ transition (reducing *c*_2_) and induced apoptosis (increasing *d*_*a*_). As treatment intensity increased, both of these fitted parameters moved in the expected direction. At high intensity (12 *μ*M concentration) the match of the model to cell cycle analysis data was not good, indicating that perhaps other transitions in the cell cycle were affected as well at this intensity. The monolayer model is thus useful for pointing out a possible inconsistency between the data and conclusions in [11] at higher intensities of treatment.

Fitting a linear model to cell cycle information for a monolayer can be done statistically. In (5) we give algebraic expressions that compute the parameters for such a model. Given the doubling time, death rate, and cell cycle analysis for an unsynchronized culture, these equations produce the remaining parameters necessary for the linear model.

Figure 6 was constructed in imitation of a similar figure in Klement et al. [2], where treatment comparisons between monolayer and spheroid cultures are given for three choices of cell line (MD22, MVB9, and CDDP-S) and treatment (adriamycin, vinblastine, and cisplatinum, resp.). The data available for the example in this paper was coarser, but the overall effect is the same. As the intensity of treatment goes up, the spheroid shows a resilience that the monolayer does not. This resilience is not due to evolved resistant strains, which were neither present in the monolayer experiment nor built into the simulations, but just to the overall anatomy and physiology of the tumor spheroid, as represented by the model. The simulations here suggest that one should expect similar reduced response to treatment intensity from nonresistant strains as well as resistant ones.

Our results give a spheroid model that not only has the correct qualitative growth behavior [4] and not only matches quantitative growth data for the control [1] but also gives qualitatively correct response to therapy in comparison with monolayer experiment [2, 11], an important new benchmark for judging the qualitative accuracy of models. The results presented here are an important step in constructing a compartment model that captures the fundamental properties of* in vivo* tumor growth. Systems of ordinary differential equations like these are quite simple compared to spatial models but have the advantage that the full force of analytic and control theoretic methods can be brought to bear on them in future work.

The ability of the model to approximate spheroid growth over time, while including cell cycle dynamics, should make it useful to the experimenter who wishes to predict the results of specific therapeutic actions on preangiogenic tumors. Its utility as a predictor fits the proposed workflow model in McGuire et al. [19]. In particular, it can help a researcher decide on a set of protocols for* in vivo* experiments.  Figure 6 indicates that, for the example given in this paper, experimenters might have difficulty distinguishing the difference in effect between 2, 4, and 8 *μ*M concentrations. It is easy to see that a more extensive data set, even for just monolayer treatments, could be coupled with this model to give more insight into what intensities of treatment are likely to produce a noticeable response.

Figure 8 shows that *c*_2_ has a big effect on long-term dynamics of the spheroid model. Near the monolayer critical value, reducing *c*_2_ has a bigger effect on final tumor size than increasing *d*_*a*_. This mathematical result highlights the importance of blocking the *G*_2_ to *G*_1_ transition as a therapeutic goal. It suggests that a less toxic therapy that blocks this transition but does not necessarily kill cells, applied over a much longer duration, could be a useful therapeutic tool. Models such as the one presented here should be part of the conversation about metronomic therapies because they identify possibilities for low level interventions which, sustained long enough, may have therapeutic effect.

An accurate model for growth of* in vitro* spheroids and their response to treatment is an important step in constructing* in silica* representations of* in vivo* tumor growth. The example presented here shows the capacity of a model to fit both qualitative and quantitative observations of spheroid growth and treatment response, give insight into the mechanisms of a particular therapy, and suggest strategies for future therapies.

## Figures and Tables

**Table 1:** Summary of cell cycle parameters for SK-N-SH monolayer culture (default parameters). Note that values for *g*_1_^*∗*^, *s* ^*∗*^, and *g*_2_^*∗*^ are adjusted slightly from Kim et al. [11] to sum to 100%. *D* is an average over several studies (refer to Ross et al. (1981), Carlsson et al. (1983), Biedler et al. (1978), Seeger et al. (1977), Barbier et al. (2001), and Smets et al. (1991)).

Cell line	SK-N-SH
Source	Kim et al. (2003)
Observed *g*_1_^*∗*^	62.87%
Observed *s* ^*∗*^	26.93%
Observed *g*_2_^*∗*^	10.20%
Observed *D*	n/a
Observed *d*_*a*_	Taken as 0

Source 2	Multiple
Observed *D*	*D* = 1.77 days

Calculated *λ*	0.40
Calculated *c*_1_	0.85
Calculated *c*_*S*_	1.59
Calculated *c*_2_	3.85

**Table 2:** Summary of treatment parameters for SK-N-SH monolayer culture. Initial conditions have total cells at 100, divided into percents given by *g*_1_^*∗*^, *s* ^*∗*^, and *g*_2_^*∗*^ for the control (see Table 1).

Cell line	SK-N-SH
Source	Kim et al. (2003)
Treatment	15-Deoxy-*PGJ*_2_ for 24 hrs
Parameter(s)	*c*_2_, *d*_*a*_
Computed final total control	147.83
Initial conditions for all runs	*g*_1_(0) = 62.87, *s*(0) = 26.93, *g*_2_(0) = 10.30, *T* = 100

Treatment intensity	2 *μ*m
Fitted parameter	*c*_2,new_ = 0.47*c*_2_, *d*_*a*,new_ = 0.60
Observed at 24 hrs % *g*_1_, % *s*, % *g*_2_, % dead	57.9%, 25.5%, 16.6%, 8.04%
Computed: % *g*_1_, % *s*, % *g*_2_	56.33%, 27.28%, 16.39%
Computed total	119.34
Computed mortality	8%

Treatment intensity	4 *μ*m
Fitted parameter	*c*_2,new_ = 0.29*c*_2_, *d*_*a*,new_ = .59
Observed at 24 hrs % *g*_1_, % *s*, % *g*_2_, % dead	51.9%, 26.4%, 21.7%, 10%
Computed: % *g*_1_, % *s*, % *g*_2_	51.34%, 26.78%, 21.88%
Computed total	109.90
Computed mortality	10.09%

Treatment intensity	8 *μ*m
Fitted parameter	*c*_2,new_ = 0.19*c*_2_, *d*_*a*,new_ = .87
Observed at 24 hrs % *g*_1_, % *s*, % *g*_2_, % dead	48.2%, 23.1%, 28.7%, 17%
Computed: % *g*_1_, % *s*, % *g*_2_	47.73%, 27.54%, 24.72%
Computed total	97.37
Computed mortality	17.03%

Treatment intensity	12 *μ*m
Fitted parameter	*c*_2,new_ = 0.001*c*_2_, *d*_*a*,new_ = 1.3
Observed at 24 hrs. % *g*_1_, % *s*, % *g*_2_, % dead	37.6%, 20%, 42.4%, 36%
Computed: % *g*_1_, % *s*, % *g*_2_	36.45%, 29.31%, 34.24%
Computed total	73.83
Computed mortality	26.25%

**Table 3:** Summary of spheroid growth data estimated from Carlsson et al. [1].

Type, cell line	(Day, reported diameter in mm), *W*	Thickness of viable cell rim at end of trial (day, *V*)
Neuroblastoma, SK-N-SH	(2, 0.19), (7, 0.21), (11, 0.27), (15, 0.32), (19, 0.35), (25, 0.39), (31, 0.41)	(31, 50–150 *μ*m)

**Table 4:** Summary of default parameters for spheroid model. Spheroid volumes and viable cell volume estimated from Table 3. Unspecified parameters from Table 1. *P* = *G*_1_ + *S* + *G*_2_. Initial conditions: *N* = 0, *Q* = 0, *G*_1_ = 2.26, *S* = .969, and *G*_2_ = .359, at time *t* = 2.

Cell line	SK-N-SH
(Time, computed volume)	(2, 3.59*∗*10^6^ *μ*m^3^)
(Time, computed volume)	(7, 4.85*∗*10^6^ *μ*m^3^)
(Time, computed volume)	(11, 10.0*∗*10^6^ *μ*m^3^)
(Time, computed volume)	(15, 17.0*∗*10^6^ *μ*m^3^)
(Time, computed volume)	(19, 22.0*∗*10^6^ *μ*m^3^)
(Time, computed volume)	(25, 31.0*∗*10^6^)
(Time, computed volume)	(31, 36.0*∗*10^6^)
(Time, computed volume of necrosis (*N*))	(31, (15.6–4.85) *∗*10^6^ *μ*m^3^)
(Time, computed volume of live cells (*T* − *N*))	(31, (20.5–31.25) *∗*10^6^ *μ*m^3^)

Chosen *s*_1_	0.0001
Fitted *c*_*q*_	0.1212
Chosen *s*_*q*_	0.0001
Fitted *s*_*n*_	61.0520
Fitted *e*	0.4898
Fitted *m*	.0528

*P* + *Q*(*t* = end)	12.65
*P* + *Q* + *N*(*t* = end)	35.94
*N*(*t* = end)	23.29
*S*(*t* = end)	2.23
*T* ^*∗*^	36.76
*N* ^*∗*^	25.59
