# Erratum to “A Model for Spheroid versus Monolayer Response of SK-N-SH Neuroblastoma Cells to Treatment with 15-Deoxy-*PGJ*_2_”

**DOI:** 10.1155/2017/6853495

**Published:** 2017-04-05

**Authors:** Dorothy I. Wallace, Ann Dunham, Paula X. Chen, Michelle Chen, Milan Huynh, Evan Rheingold, Olivia Prosper

**Affiliations:** ^1^Department of Mathematics, HB 6188, Dartmouth College, Hanover, NH 03768, USA; ^2^Department of Mathematics, University of Kentucky, Lexington, KY 40506, USA

In the article titled “A Model for Spheroid versus Monolayer Response of SK-N-SH Neuroblastoma Cells to Treatment with 15-Deoxy-*PGJ*_2_” [1], the fifth author's affiliation was incorrect. The correct affiliation is shown above.

## References

[1] Wallace D. I., Dunham A., Chen P. X. (2016). A model for spheroid versus monolayer response of SK-N-SH neuroblastoma cells to treatment with 15-deoxy-*PGJ*_2_. *Computational and Mathematical Methods in Medicine*.

